# Agenesis of the dorsal pancreas presenting with diabetic ketoacidosis – a case report and literature review

**DOI:** 10.1186/s12902-019-0449-1

**Published:** 2019-11-11

**Authors:** Tian Yang, Xudan Yang, Luping Wang, Jun Mo

**Affiliations:** 1 Department of Endocrinology, Fourth Affiliated Hospital, School of Medicine, Zhejiang University, Yiwu, 322000, Shangcheng Road N1, Yiwu, Zhejiang, China; 2 Department of Internal Medicine, Jinhua Municipal Central Hospital of, Zhejiang University, Jinhua, Zhejiang, 321000 China; 3 Department of Neurosurgery, Fourth Affiliated Hospital, School of Medicine, Zhejiang University, Yiwu, Zhejiang, 322000 China

**Keywords:** Agenesis of the dorsal pancreas, Diabetic ketoacidosis, Diabetes mellitus, C-peptide release test

## Abstract

**Background:**

Agenesis of the dorsal pancreas (ADP) is clinically rare, and it is usually accompanied by abdominal pain. Various disorders of glucose metabolism associating with ADP have been reported, but there are only two studies reporting a correlation between ADP and DKA in English literature.

**Case presentation:**

We present a case of a patient with ADP accompanied by abdominal pain and diabetic ketoacidosis as the initial clinical presentation. A 30-year-old man presented with a 3-month history of recurrent onset of persistent mild epigastric pain, which worsen when eating. Laboratory tests revealed metabolic acidosis, hyperglycemia, and ketonuria. Phase contrast CT and MRCP showed the absence of the body and tail of the pancreas, as well as the dorsal pancreatic duct. The C-peptide release test indicated β-cell dysfunction. A combination therapy of insulin, pancreatic enzyme supplements, and mosapride citrate were administrated and the pain gradually resolved.

**Conclusions:**

As glucose metabolism disorders can vary across different individuals, we advise clinicians to consider the diagnosis of ADP for a patient who presents with a glucose metabolism disorder accompanied by abdominal pain, pancreatitis or steatorrhea.

## Background

Agenesis of the dorsal pancreas (ADP) is a rare congenital anomaly caused by the failure of the dorsal pancreatic bud to develop the body and tail of the pancreas during embryological development [1]. A key clinical manifestation of ADP is abdominal pain, although ADP often associates with hyperglycemia as a result of β-cell dysfunction and insulin deficiency [2]. However, there are only two studies reporting a correlation between ADP and DKA in English literature [3, 4]. Here, we present a third case of a patient with ADP accompanied by abdominal pain and DKA.

## Case presentation

A 30-year-old man referred to our hospital presented with a 3-month history of recurrent onset of persistent mild epigastric pain, which worsen when eating. The patient took a lot of sugary beverages one week before his admission to the hospital. He had no history of diarrhea, dry mouth, polyuria, polydipsia, weight loss, and gastrointestinal disease. The family history was noncontributory. His mother died of gynecological cancer at age 50. His father had no history of hyperglycemia or chronic abdominal pain, and the abdominal CT scan showed a normal pancreas. His only younger sister had no special medical history as well. A physical examination revealed that the patient was in good shape (body mass index 22.7 kg/m^2^). He was conscious but dehydrated. He had a soft but tender abdomen, and his heart and lung functions were normal. His vital signs were also normal.

Laboratory tests (Table 1) revealed metabolic acidosis with an arterial blood pH of 7.3 and a base excess of − 8.9 mmol/L. The random plasma glucose level was 576 mg/dL, with urinalysis revealing glycosuria and ketonuria. The glycated hemoglobin (HbA1c) level was 147 mmol/mol, and the serum lactic acid level was within normal range. Levels of carcinoembryonic antigen and cancer antigen 199 were also within normal ranges. The results of liver function, serum amylase, lipase, C-reactive protein, and microalbuminuria tests, as well as the 24-h urine protein level, were within normal ranges. The patient was negative for the glutamic acid decarboxylase antibody, islet cell antibody, and insulin autoantibody. The patient was diagnosed with DKA and received standard treatment for the condition, which included intravenous fluids, insulin therapy, and potassium replacement.

**Table 1:** Laboratory results of this patient

	Results	Reference Range	Units
White blood cell counting	8.7	3.5–9.5	10^9^/L
Neutrophils	50.1	40–75	%
C reactive protein	0.3	0–8	mg/L
Serum bilirubin	11.4	5.1–19.0	μmol/L
Serum albumin	37.1	40–55	g/L
Serum alkaline phosphate	48	35–100	U/L
Serum aspartate	16	13–35	U/L
Serum amylase	75	35–135	U/L
Fasting plasma glucose	576	70–110	mg/dL
HBA1c	147	16–42	mmol/mol

**Fig. 1:**
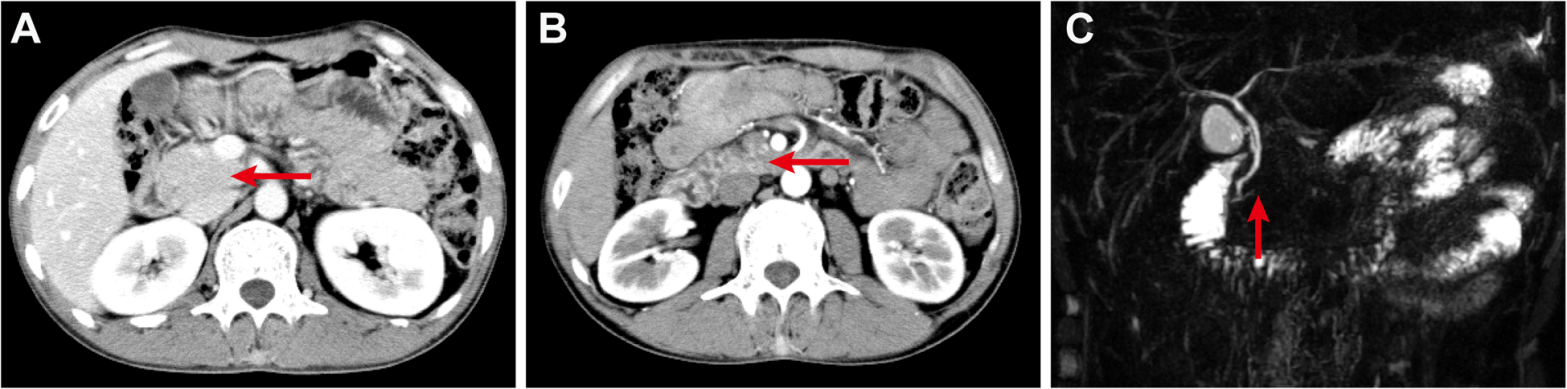
Contrast abdominal computed tomography scan showed the pancreatic head (**a**, red arrow), whereas the pancreatic body and tail are absent (**b**, red arrow). Magnetic resonance cholangiopancreatography demonstrated the absence of the dorsal pancreatic duct (**c**, red arrow)

DKA resolved gradually after insulin therapy, but the abdominal pain continued. Additional phase contrast CT of the abdomen was performed and revealed an enlarged pancreatic head (Fig. 1A), without the body and tail of the pancreas (Fig. 1B). A further investigation of MRCP revealed the absence of the dorsal pancreatic duct and a short duct of Wirsung running into the major papilla (Fig. 1C). On the basis of these findings, a diagnosis of complete ADP was evident, and we believed that the pain was due to dysfunction of the pancreas. Low-fat diet was recommended, and pancreatic enzyme supplements as well as mosapride citrate were given with meals to facilitate the digestive process. The pain gradually resolved and went away in 7 days after the treatment.

A standard mixed-meal tolerance test was performed one month later to evaluate β-cell function. The fasting C-peptide level was 0.05 ng/mL, and the postprandial C-peptide levels at 1, 2, and 3 h were 0.05, 0.07, and 0.06 ng/mL (normal range, 1.1–4.4 ng/mL), whereas the fasting plasma glucose level was 261 mg/dL, and the postprandial glucose levels at 1, 2, and 3 h were 433, 455, and 433 mg/dL, respectively. According to the patient’s medical history and laboratory results, we speculated that the sugary beverages might resulted in high blood glucose, which may contribute to DKA in this patient. According to the ADA’s standard of classification and diagnosis of diabetes, the diagnosis of “Specific types of diabetes due to other causes” was established [1, 2]. The patient received insulin therapy (insulin glargine 12 units at bedtime and biosynthetic human insulin 16 units with meals) and was followed up.

## Discussion and conclusions

The pancreas develops from the ventral and dorsal buds, which fuse during the seventh week of gestation. The ventral bud gives rise to the uncinate process, post-inferior portion of the head, and Wirsung duct, whereas the dorsal bud, which drains into the minor papilla through the Santorini duct, gives rise to the upper head, body and tail [5]. Monogenic mutations in insulin promoter factor 1 [6], pancreas associated transcription factor 1 [7], and transcription factor-2 / hepatocyte nuclear factor-1 homeobox B [8] have been reported to associate with pancreatic agenesis, multigenic traits are likely to contribute to this disorder. However, one limitation should be noted that we didn’t have genetic analysis in the presented case as the patient refused DNA sequencing test.

We reviewed the articles published between January 2008 and August 2019 and 75 cases of ADP were identified. Of the 75 cases, 53 cases that had been reported by Cienfuegos were excluded from the study [9]. Clinical presentation, pancreas imaging, and gene mutation results were extracted and summarized (Table 2). Although the majority of ADP patients are asymptomatic, abdominal pain is the most common reported symptom. The abdominal pain may contribute to the dysfunction of the sphincter of Oddi and/or chronic pancreatitis accompanied by an elevated pancreatic intra-ductal pressure [10]. In this case, we at first believed that the pain was caused by DKA. However, the abdominal pain continued after rectifying the DKA, indicating that the abdominal pain was caused by ADP.

**Table 2:** Characteristics of the selected studies

Studies	Clinical presentation	Pancreas imaging	Gene mutation
Devarbhavi PK [3]	Diabetic ketoacidosis	Short pancreatic tail	Not assessed
Sohn TS [4]	Severe hypertriglyceridemia, and acute pancreatitis	Pancreas tail and dorsal pancreas duct were not visualized	Not assessed
Caetano LA [5]	Maturity onset diabetes of the young	Caudal pancreatic agenesis	Heterozygous variant in PDX1
Caetano LA [5]	Impaired glucose tolerance	Short pancreas tail	Heterozygous variant in PDX1
Cienfuegos JA [9]	DM, mucinous cysts and chronic calcific non-alcoholic pancreatitis	Mucinous cysts	Not assessed
Liang K [14]	DM	Normal shape of pancreatic head	Not assessed
Erotokritou A [17]	DM, nonspecific abdominal symptoms	Neuroendocrine tumor	Not assessed
Kawasaki S [19]	Pancreatitis, Peutz-Jegher syndrome	Normal shape of pancreatic head	Not assessed
Alexander E [21]	Pancreatic head cancer, obstructive jaundice	Hypo-vascular lesion in the head	Not assessed
Suh PS [22]	DM	Cystic mass lesion	Not assessed
Suh PS [22]	DM	Calcified cystic mass	Not assessed
Riguetto CM [23]	DM, heterotaxy syndrome	Enlarged pancreas head	Not assessed
Sonkar SK [24]	DM, recurrent loose stool and abdominal pain	Agenesis of dorsal pancreas	Not assessed
Jain A [25]	DM, recurrent upper abdominal pain, fatigue	Pancreatic body and tail were not visible in MRCP	Not assessed
Rodrigues P [26]	Neuroendocrine tumor	Nodular-lesion on pancreas head	Not assessed
Chhabra P [27]	Epigastric pain aggravated by meals	Normal shape of pancreatic head	Not assessed
Mustafa K [28]	DM, polysplenia, Kartagener syndrome, polycystic kidney disease.	Hypertrophied ventral pancreas	Not assessed
Kabnurkar R [29]	Carcinoma of tongue	Normal shape of pancreatic head	Not assessed
Saikaly E [30]	Mucinous adenocarcinoma and cystic teratoma	Complex cystic lesion	Not assessed
Shahzad R [31]	No	Agenesis of dorsal pancreas	Not assessed
Robert AP [32]	Right iliac fossa pain	Normal shape of pancreatic head	Not assessed
Nassif S [33]	Pancreatic neuroendocrine tumor, endometrial stromal sarcoma	Mass at the neck of the pancreas	Not assessed

Patients with ADP may also present with disorders of glucose metabolism, such as insulin-dependent diabetes, high-fasting blood glucose levels, and non-insulin-dependent diabetes [11]. According to the published reports, approximately 50% of patients with ADP also have concomitant hyperglycemia [12]. Although β-cell dysfunction is often indicative of hyperglycemia, there are only two studies reporting a correlation between ADP and DKA [3, 4]. Four cases of ADP, including the present one, had reported C-peptide test results, three of which showed low levels of fasting and postprandial C-peptide associated with β-cell dysfunction [13, 14], and one case showed detectable C-peptide level of 0.47 nmol/L [3]. Therefore, low insulin levels underlie most of the glucose metabolism disorders, as islets and β-cells are located in the tail of the pancreas [15, 16]. Previous studies have reported variations in the severity of high-fasting blood glucose disorders and insulin-dependent diabetes [12, 17], indicating that there are many degrees of β-cell dysfunction in patients with ADP.

Other abdominal symptoms including pancreatitis and steatorrhea have also been reported [18, 19]. The reported incidence of pancreatitis was 30% [12], but it is unclear whether the high frequency of pancreatitis in ADP patient was due to the requirement of imaging procedure for patient with pancreatitis. Steatorrhea in ADP patient was due to exocrine pancreatic insufficiency. Although the prevalence is much less common, most of the cases had concomitant hyperglycemia [18].

Imaging modalities are essential in the diagnosis of ADP, with ultrasonography as the most commonly used approach for evaluating abdominal pain and other abdominal symptoms [20]. However, interference from the superimposed gas in the stomach and duodenum limits its usefulness in the detection of pancreatic anomalies [14]. Both CT and MRCP are reliable modalities to confirm the absence of the body and tail of the pancreas and to differentiate this condition from other disorders such as periportal lymphadenopathy and anatomic variations. ERCP and MRCP can also be used to confirm the absence of the dorsal duct system. In summary, MRCP is a noninvasive approach with no risk of exposure to radiation, and we recommend it as the first choice for patients with ADP.

As glucose metabolism disorders can vary across different individuals, we advise clinicians to consider the diagnosis of ADP for a patient presenting with a glucose metabolism disorder accompanied by abdominal pain, pancreatitis or steatorrhea.

Informed consent was obtained from this patient for publication of this case history and associated images were provided.

## Data Availability

The CT scan and MR imagines were not shared publicly as they contained identifying/confidential information of the patient.

## Abbreviations

ADP: Agenesis of the dorsal pancreas
CT: Computed tomography
DKA: Diabetic ketoacidosis
Dm: Diabetes mellitus
ERCP: Endoscopic retrograde cholangiopancreatography.
MRCP: Magnetic resonance cholangiopancreatography

## References

[1] Association AD (2019). Classification and diagnosis of diabetes: standards of medical care in diabetes-2019. Diabetes Care.

[2] Buzzetti R, Zampetti S, Maddaloni E (2017). Adult-onset autoimmune diabetes: current knowledge and implications for management. Nat Rev Endocrinol.

[3] Devarbhavi PK, Bhagwat KA, Patil TCR, Murthy V, Chakravarthi S (2014). Dorsal agenesis of pancreas manifesting clinically as diabetic ketoacidosis: a rare case study. Ann Res & Rev in Biol.

[4] Sohn TS, Kim HH, Seo W, Lee KP, Seok H, Son HS. Diabetic ketoacidosis, severe hypertriglyceridemia, and acute pancreatitis in a patient with agenesis of the dorsal pancreas. *Endocr Rev* 2018, 39(2):Supplement 1.

[5] Caetano LA, Santana LS, Costa-Riquetto AD, Lerario AM, Nery M, Nogueira GF, Ortega CD, Rocha MS, Jorge AAL, Teles MG (2018). PDX1-MODY and dorsal pancreatic agenesis: new phenotype of a rare disease. Clin Genet.

[6] Stoffers DA, Zinkin NT, Stanojevic V, Clarke WL, Habener JF (1997). Pancreatic agenesis attributable to a single nucleotide deletion in the human IPF1 gene coding sequence. Nat Genet.

[7] Sellick GS, Barker KT, Stolte-Dijkstra I, Fleischmann C, Coleman RJ, Garrett C, Gloyn AL, Edghill EL, Hattersley AT, Wellauer PK (2004). Mutations in PTF1A cause pancreatic and cerebellar agenesis. Nat Genet.

[8] Haumaitre C, Barbacci E, Jenny M, Ott MO, Gradwohl G, Cereghini S (2005). Lack of TCF2/vHNF1 in mice leads to pancreas agenesis. Proc Natl Acad Sci U S A.

[9] Cienfuegos JA, Benito A, Rotellar F (2017). Agenesis of the dorsal pancreas associated with mucinous cysts and chronic calcific non-alcoholic pancreatitis. Rev Esp Enferm Dig.

[10] Uygur-Bayramicli O, Dabak R, Kilicoglu G, Dolapcioglu C, Oztas D (2007). Dorsal pancreatic agenesis. JOP.

[11] Shimodaira M, Kumagai N, Sorimachi E, Hara M, Honda K (2012). Agenesis of the dorsal pancreas: a rare cause of diabetes. Intern Emerg Med.

[12] Schnedl WJ, Piswanger-Soelkner C, Wallner SJ, Reittner P, Krause R, Lipp RW, Hohmeier HE (2009). Agenesis of the dorsal pancreas and associated diseases. Dig Dis Sci.

[13] Du J, Xu GQ, Xu P, Jin EY, Liu Q, Li YM (2007). Congenital short pancreas. Chin Med J.

[14] Liang K, Ou X, Huang X, Lan Q (2018). Agenesis of the dorsal pancreas: a rare cause of insulin-dependent diabetes without abdominal pain: case report. Medicine (Baltimore).

[15] Rahier J, Goebbels RM, Henquin JC (1983). Cellular composition of the human diabetic pancreas. Diabetologia.

[16] Wittingen J, Frey CF (1974). Islet concentration in the head, body, tail and uncinate process of the pancreas. Ann Surg.

[17] Erotokritou A, Gerharz CD, Sagir A (2018). Agenesis of dorsal pancreas associated with pancreatic neuroendocrine tumor: a case report and review of the literature. J Med Case Rep.

[18] Doxey BW, Jackson WD, Adler DG (2008). A unique presentation: dorsal agenesis of the pancreas manifesting as pancreatic exocrine insufficiency in the absence of diabetes mellitus in an 8-year-old boy. Dig Dis Sci.

[19] Kawasaki S, Itoi T, Iwasaki E, Hosoe N, Ogata H, Kanai T (2016). Successful pancreatic duct stent placement for recurrent pancreatitis in a patient with polysplenia with agenesis of the dorsal pancreas and peutz-jeghers syndrome. Intern Med.

[20] Vijayaraghavan SB, Gouru S, Senthil S (2013). Sonographic features of agenesis of dorsal pancreas. Indian J Radiol Imaging.

[21] Julianov AE, Saroglu AS (2019). Pancreatic head cancer in a patient with complete agenesis of dorsal pancreas. Hepatobiliary Surg Nutr.

[22] Suh PS, Lee JH, Yu JS, Hee Kim J, Kim B, Kim HJ, Huh J, Kim JK, Lee D (2019). Three cases of pancreatic pseudocysts associated with dorsal pancreatic agenesis. Radiol Case Rep.

[23] Riguetto CM, Pelichek S, Moura Neto A (2019). Heterotaxy syndrome with agenesis of dorsal pancreas and diabetes mellitus: case report and review of the literature. Arch Endocrinol Metab.

[24] Sonkar SK, Kumar S, Singh NK. Agenesis of dorsal pancreas in a young adult: a rare cause of diabetes mellitus. *BMJ Case Rep*. 2018;(pii):bcr-2017–223301. 10.1136/bcr-2017-223301 PMC5847835 29444795

[25] Jain A, Singh M, Dey S, Kaura A, Diwakar G (2017). A rare case ofcomplete agenesis of dorsal pancreas. Euroasian J Hepatogastroenterol.

[26] Rodrigues P, Oliveira RC, Oliveira CM, Cipriano MA. Neuroendocrine tumour in pancreatic dorsal agenesis: a rare association. *BMJ Case Rep*. 2017;(pii):bcr-2017–221999. 10.1136/bcr-2017-221999 PMC5665190 29066640

[27] Chhabra P, Brar R, Bhasin DK (2017). Unusual case of abdominalpain: finding the missing part. Gastroenterology.

[28] Demir MK, Furuncuoglu Y (2017). Coincidence of polysplenia, kartagener syndrome, dorsal pancreas agenesis, and polycystic kidney disease in an adult. Eurasian J Med.

[29] Kabnurkar R, Rokade ML, Bandekar K, Kamat N (2017). Incidentally detected agenesis of dorsal pancreas on PET/CT: case report and review of literature. Indian J Nucl Med.

[30] Saikaly E, El Asmar A, Abi Fadel F, Aoun M, El Rassi Z (2017). Agenesis of the dorsal pancreas associated with mucinous adenocarcinoma and cystic teratoma: a case report and literature review. Clin Case Rep.

[31] Shahzad R, Shahid AB, Mirza ZR, Anees A (2016). Isolated dorsal pancreatic agenesis. J Coll Physicians Surg Pak.

[32] Robert AP, Iqbal S, John M (2016). Complete agenesis of the dorsal pancreas: a rare clinical entity. Int J Appl Basic Med Res.

[33] Nassif S, Ponchiardi C, Sachs T (2016). Pancreatic neuroendocrine tumor in the setting of dorsal agenesis of the pancreas. Case Rep Gastrointest Med.

